# Role of cellular reprogramming and epigenetic dysregulation in acquired chemoresistance in breast cancer

**DOI:** 10.20517/cdr.2018.11

**Published:** 2019-06-19

**Authors:** Logeswari Ponnusamy, Prathap Kumar S. Mahalingaiah, Yu-Wei Chang, Kamaleshwar P. Singh

**Affiliations:** ^1^Department of Environmental Toxicology, The Institute of Environmental and Human Health (TIEHH), Texas Tech University, Lubbock, Texas 79409, USA.; ^2^Metabolism and Safety, Zoetis, Kalamazoo, Michigan 49007, USA [current affiliation].; ^3^Preclinical Safety, AbbVie, North Chicago, Illinois 60085, USA [current affiliation].

**Keywords:** Chemoresistance, cellular reprogramming, DNA methylation, histone modifications, breast cancer, epithelial to mesenchymal transition, cancer stem cell

## Abstract

Acquired resistance to chemotherapy is a major limitation in clinical treatment for breast cancer. Accumulating evidence from *in vitro*, *in vivo* and clinical studies suggest that acquired chemoresistance is progressive, multifactorial and involve genetic and epigenetic aberrations. Among various mechanisms that contribute to chemoresistance, cellular reprogramming has extensively been implicated in breast cancer resistance lately. Cellular reprogramming events such as acquisition of epithelial to mesenchymal transition (EMT) and cancer stemness (CSCs) not only provide cancer cells with reversible phenotypic plasticity and survival advantage against cytotoxicity but also leads to aggressiveness, metastasis, clinical resistance, tumor recurrence and poor survival. The transient and reversible nature of cellular reprogramming processes and their controlled interaction with epigenetic regulatory complexes strongly support the involvement of dynamic epigenetic regulatory network in governing the cellular reprogramming and associated acquired chemoresistance. Further, epigenetic modulations are also gaining interest as promising interventions addressing the cancer cell reprogramming machinery to overcome acquired chemoresistance. This review discusses the previous reports and our recent findings that lead to current understanding of epigenetic dysregulation dictating the cellular reprogramming processes such as acquisition of EMT and CSCs phenotype and how they co-ordinate to establish acquired drug resistance in breast cancer.

## Introduction

Breast cancer is the leading cause of cancer-related death in women globally. Various treatment approaches such as local therapy (radiation) and systemic therapy including hormones, chemotherapeutic agents and targeted therapeutic approaches are commonly used in breast cancer patients^[1]^. Chemotherapy, either as a single or combination therapy, is the primary choice of treatment for advanced stage breast cancer patients. But, non-responsiveness or resistance development to chemotherapy is very common and often result in treatment failure, hence remains as the major hurdle for the effective clinical management and disease-free survival of breast cancer patients^[2]^. Chemoresistance is a dynamic and complex process that can be of inherent or acquired during the therapy. Highly heterogenic origin of breast cancer with high degree of variability in differentiation impacts the chemotherapy sensitivity and resistance development^[3]^. Besides presenting as cross-resistance to combination therapy, prolonged exposure of breast cancer patients to a single drug may also result in tolerance against multiple structurally and mechanistically diverse, unexposed drugs that is commonly referred as multidrug resistance^[2]^.

Several complex regulatory signaling mechanisms dynamically cross-talk to initiate, establish and maintain tolerance/resistance to different chemotherapeutic agents in breast cancer. At large, both pharmacokinetics and pharmacodynamic mechanisms and their reinforcement have been implicated in acquired chemoresistance^[4,5]^. Pharmacokinetic mechanisms such as aberrant expression of drug transporters (reduced drug influx or increased efflux), drug compartmentalization in intracellular organelles and altered drug metabolism (inactivation and detoxification) would contribute to tolerance development. Similarly, pharmacodynamic mechanisms mainly including altered targets expression (both qualitative/quantitative; over/under expression) and function, genomic instability, mutation of target enzymes, failure to undergo apoptosis, impaired DNA repair, oxidative stress, microenvironment and cellular reprogramming leading to phenotypic plasticity including epithelial to mesenchymal transition (EMT) and acquisition of cancer stem cells (CSCs) characters may eventually contribute to tolerance to cytotoxicity and acquired resistance development^[2,4,5]^.

**Figure 1:**
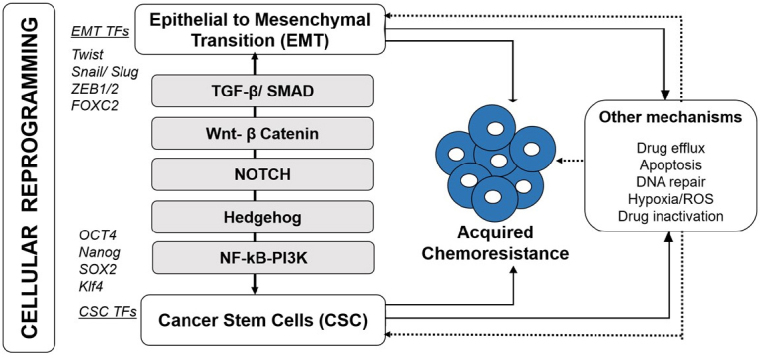
Acquired chemoresistance in breast cancer involves cellular reprogramming. Induction of epithelial to mesenchymal transition (EMT) and cancer stem cell (CSC) facilitate resistance development and tumor recurrence. These processes employ EMT and CSC-specific Transcription factors (TFs) and activated through key signaling pathways including TGF-β/SMAD, Wnt-β catenin, Notch, Hedgehog (Hg) and NF-κB/PI3K. Other mechanisms such as increased drug efflux, apoptosis, DNA repair and other microenvironmental changes in addition to their direct involvement in resistance development, indirectly co-activated/ involved during cellular reprogramming (EMT-CSC)-mediated chemoresistance

In the recent years, among all the potential mechanisms which contribute to acquired chemoresistance, increased attention is focused on role of cellular reprogramming with acquisition of EMT and CSC phenotype [Figure 1]^[6-8]^. In addition, mounting evidence on the role of nuclear interaction of reprogramming factors such as transcription factors (TFs) of EMT and stemness with regulatory protein complexes within epigenetic landscape further establish the role of epigenetic changes in cellular reprogramming contributing to acquired chemoresistance^[7,9,10]^. Moreover, epigenetic modulations are also gaining interest as promising interventional approaches targeting the cancer cell reprogramming machinery to overcome acquired chemoresistance in breast cancer^[8,11]^. In this review, we are mainly discussing previous reports and our recent findings on epigenetic modifications associated with the regulation of cellular reprogramming processes such as acquisition of EMT and CSCs phenotype during the acquisition of drug resistance.

## Cellular reprogramming

### Epithelial to mesenchymal transition

EMT is a conserved, highly complex cellular process that is tightly regulated by various signaling pathways and transcription factors and controlled by epigenetic reprogramming^[9]^. During EMT process, epithelial cells lose their epithelial features such as cell-cell adhesion and polarity while acquiring the characteristics of mesenchymal cells, such as increased motility, aggressiveness and invasion. These morphological changes are accompanied with changes of loss and gain of functional proteins associated with epithelial and mesenchymal properties respectively. EMT- inducers are group of transcription factors that comprises of the Snail (Snail/Slug), ZEB (ZEB1/2), and basic helix-loop-helix (TWIST1/2, TCF3) proteins that have specific target genes involved primarily in EMT process and in recruiting epigenetic regulatory units to exerts their downstream functions^[9]^. It is important to note that these EMT-TFs also co-ordinates with CSC-TFs to induce stemness.

Besides the embryonic development, wound healing and fibrosis, preferential activation of EMT is well established in tumor metastasis and during chemotherapy to provide growth advantage that drive the clinical resistance^[12]^. The degree of EMT and the level of differentiation through heterogeneity may also impact the response of the metastatic tumor cells to chemotherapy. EMT-driven chemoresistance may potentially occur through the common signaling processes that activate not only the EMT but also other survival pathways including stemness induction^[13,14]^.

### Cancer stem cells

CSCs are sub-population of cells within tumor that possess tumorigenicity as well as capacity to self-renew after acquiring the genetic and epigenetic modifications needed for adaptation and clonal growth during the metastatic process and chemotherapy cycles. There are different theories debating the origin of breast cancer stem cells. Available experimental evidence posits breast cancer stem cells to be derived from mammary stem cells, mammary progenitor cells or differentiated mammary cells^[15]^. Functional mutations accrued during the quiescent state of the mammary stem cells or progenitor cells induce oncogenic transformation and perpetrate malignancy^[15,16]^. Similarly, differentiated mammary cells, upon environmental exposure such as chemotherapy, de-differentiate to induce *de novo* stem cell traits^[15,17]^. These CSCs are epigenetically unstable and tumorigenic in immunodominant animal models^[18,19]^. Induction and maintenance of the stemness are regulated by several key TFs including Octomer binding transcription factor 4 (OCT4), Nanog homeobox (Nanog), Krupple-like factor 4 (Klf4) and Sex determining region Y box-2 (SOX2). CSCs express distinctive cell surface markers such as CD24, CD29, CD34, CD38, CD44, CD61, CD90, CD133, CD166, EpCAM, Epithelial antigen (ESA) and CXCR4, and selective ATB-binding Cassette (ABC) transporters such as BCRP, and MRP5; selective expression of these markers differentiate the cancer subtypes^[19-21]^.

Early observations postulated that sub-population of cells within tumor introduce heterogeneity that impact the drug response. Such heterogenic phenotypes are closely related to stem cells that express specific markers, and, as compared to non-CSC cells, exhibit reduced sensitivity to antitumor agents, refractory to therapy and renders reversible drug tolerant state^[3,22]^. In line, chemotherapy exert selective pressure that not only enrich for CSCs but also induce drug tolerant stem cells which further repopulate the treated tumor and lead to recurrence and aggressiveness. Mounting evidence support that heterogeneity-associated phenotypic plasticity facilitate acquired resistance development^[3,23]^.

Recent studies have significantly increased our understanding of the tumorigenic CSCs and molecular mechanisms that foster their growth and maintenance with protection against chemotherapeutics. Stem-like cancer cells accumulate mutations and gain survival advantage during therapy through several mechanisms including their high self-renewal potential, metabolically slow/quiescence non-cycling state, enhanced expression of drug transporters, anti-apoptotic proteins, and proficient or augmented DNA damage repair process^[21]^. Breast cancer stem cells, with signature putative markers such as CD44-high, CD24-low, ESA+ and ALDH1-high, are negatively associated with disease free survival and apparently, resistance to several anticancer agents^[20,24,25]^.

### Co-orchestration of EMT-CSC signaling is key in acquired chemoresistance in breast cancer

Regardless of the distinctive mechanisms through which EMT and CSCs phenotype arise, it is convincing that they share common regulatory mechanisms and their cross-talk is key to initiate metastasis, chemoresistance, and tumor recurrence [Figure 1]^[13,14]^. Important evidence provided by Mani *et al*.^[26]^, demonstrating the ability of EMT to induce cells with stemness laid the basis for establishing the EMT-CSC shared relationship. Since then several studies have uncovered various mechanisms and pathways involved in these two mutual processes and how they provide survival advantage to reinforce the evasion from therapy and cause resistance in breast cancer^[19,21,25]^. While EMT signaling renders acquisition and maintenance of stem cell characteristics, CSCs cells use EMT process for metastasis and to evade chemotherapy response^[14]^. Recent reports have reviewed the mechanisms underpinning EMT and CSC in chemoresistance and highlighted several examples of laboratory and clinical studies indicating the ineffectiveness of radiation, conventional chemotherapy and advanced targeted therapies towards EMT and CSCs^[13,21]^.

EMT and CSC shares common signaling pathways to orchestrate the cellular function in terms of tumor maintenance, metastasis and therapy resistance^[14]^. Four primary signaling pathways that mediate acquired breast cancer resistance through concerted EMT-CSC activation [Figure 1] are described below.

#### TGF-β-SMAD-dependent and SMAD-independent signaling pathways

Transforming growth factor-beta (TGF-β) is a key regulator controlling cell growth, differentiation and anti-apoptosis and induce mesenchymal-derived cells to set forth EMT process, migration and invasion and chemoresistance^[27,28]^. Sequentially, TGF-β signaling further upregulate CSC-inducing factors including Nanog, SOX and support CSC maintenance either directly or through microenvironmental changes^[26,28]^. Activation of TGF-β trigger SMAD-family proteins and their interaction with EMT-TFs to repress E-cadherin, an initial step in the EMT process^[14]^. In turn, activation of SMAD leads to elevation of SOX4/SOX2 expression that enable quiescence and cell cycle arrest in CSCs to protect cells from cytotoxicity. However, another study found TGF-β mediated elevated SOX4 is indeed important for EMT and mesenchymal phenotype^[29]^. TGF-β also involves SMAD-independent signaling to induce EMT and cancer resistance through distinct tyrosine kinase receptor pathways such as PI3K/Akt and mTOR^[27,28,30]^. TGF-β induced invasion during EMT process is orchestrated along with activation of anti-apoptotic signaling including PI3K/Akt and NF-κB pathways thus apoptosis is prevented during this process^[27]^. Though Akt transduction and associated nuclear localization of FOXO3 are known to be involved in origin of hematopoietic stem cells^[28]^, function of SMAD-independent TGF-β signaling in breast cancer stem cell induction is not yet clearly understood. Moreover, elicitation of TGF-β modulates Wnt and NF-κB signaling to support vimentin-dependent mesenchymal state, enrich CSCs, and maintain stemness as well as tolerance^[14,27]^. TGF-β mediated EMT-CSC-based resistance has been evident from various studies. For example, doxorubicin resistant MCF-7 breast cancer cells acquired signature mesenchymal and invasive phenotype. This aggressive phenotype accompanied with Snail 1-mediated loss of E-cadherin and ESR, estrogen independency, tolerance to TNF-mediated apoptosis and concurrent alteration in TGF-β and NF-κB signaling^[20,31]^.

#### Wnt-β-catenin signaling

Among all the EMT-CSC-shared signaling pathways, Wnt/β-catenin signaling is crucial for EMT and proliferation and self-renewal of normal as well as cancer stem cells^[32,33]^. Induction of Wnt signaling modulates β-catenin stability and its nuclear translocation, to recruit histone modifying co-activators including HAT CBP/p300 and BRG1 to activate downstream transcription of various targets including Snail, Twist, Slug, ZEB1, vimentin, fibronectin, and MMPs to promote EMT and associated migration^[34]^. Wnt signaling not only induce EMT-TFs such as Slug and Twist but also shown to be highly activated in mammary stem cells contributing to their increased potential for self-renewal^[32,35]^. Through transcriptional regulation of MDR1 gene, Wnt/B-catenin signaling supports emergence of chemoresistance with acquisition of EMT phenotype^[33]^. Wnt antagonist secreted frizzled-related protein 1 (SFRP1) known to sensitize breast CSCs to doxorubicin/cisplatin-induced apoptosis^[36]^. Besides, depletion of Wnt signaling abrogated stem cell sub-population with CD44-high/ CD24-low, and ALDH1 markers and decreased metastatic potential *in vivo*^[32]^. Chemotherapy resistant and non-responsive breast cancer cells found to have elevated anti-apoptotic protein Survivin which is also a Wnt-β catenin target and has been linked to transient acquisition of EMT and stemness^[37]^.

#### Hedgehog signaling

Hedgehog (Hh) signaling have been shown to be de-regulated in various solid tumors and associated with EMT and stemness^[14]^. Activated Hh signaling increases Snail expression to repress E-Cadherin and to promote EMT and stemness which may eventually contribute to chemoresistance phenotype^[38,39]^. Hh signaling pathway also known to interact with Wnt-β-catenin signaling to promote carcinogenesis and cancer aggressiveness. This interaction of signaling can modulate both EMT and CSC pathways through elevating key TFs (such as Snail, Slug, ZEB1, ZEB 2, TWIST2 and FOXC2) and stem cell markers (such as BMI1, CD44 and CD133)^[38]^. Enrichment of aberrant Hh signaling mechanisms in aggressive, metastatic and chemoresistant breast cancers types lead to low levels of E-cadherin and increased expression of FOXC2, SIP1, Snail, and Twist, vimentin, fibronectin and N-cadherin^[14,24,38]^. Modulation of these proteins sensitize resistant cancer cells to chemotherapy which further establish the role of Hh signaling in chemoresistance.

#### Notch signaling

Notch signaling influences cell proliferation, differentiation and dictates cell fate and apoptosis. Notch signaling is regulated through Notch receptors (1-4) and ligands such as Delta-like ligand (DDL 1/3/4) and Jagged 1/2^[40]^. Notch signaling co-operates with transcription factors of EMT (Snail and Slug) and stemness (SOX2, Nanog and OCT4) and facilitate acquisition of both EMT and stemness^[14,40]^. Notch, coupled with TGF-β, induces Slug expression to mediate EMT^[40]^. Notch signaling also regulates different target genes related to CSCs and mediate chemoresistance^[41,42]^. For example, IL-6, a Notch target gene supports self-renewal potential of CSCs and Notch-mediated activation of PKB protect cells from apoptosis to induce resistance^[41]^. Deactivation of Notch signaling kill progenitor cells that are similar to CSCs which further supports the role of Notch in stemness and associated chemoresistance. MDR1 is highly expressed in CSCs while Notch signal coupled with NF-κB and associated PI3K/Akt activation regulate MRP2 transporter that favors maintenance of stemness^[20]^.

Currently, there is no refined method to differentiate tumor-derived CSCs from EMT-induced CSCs. However, growing body of literatures and discovery of advanced and new selection markers direct toward the cross talk between EMT and CSC in therapy resistance. Several *in vitro*, *in vivo* and clinical studies on various solid tumors including breast carcinoma emphasized the co-existence of EMT and CSC markers indicating the relationship between the activation of EMT leading to stemness^[13,24,26]^. Nevertheless, the order of occurrence between EMT and CSC in acquired chemoresistance is still debatable, however, accumulating evidences support that the epigenetic machinery regulating the EMT-CSC co-ordination^[6-8,43]^. Current understanding of the reversible nature of the EMT and phenotypic plasticity of CSC cells and their co-existence in resistance induction together asserts the notion of reversible epigenetic changes.

## Epigenetic regulation of acquired chemoresistance

Tumorigenesis involves cellular reprogramming that are known to be modulated through epigenetic deregulation, thus, it is more conceivable that epigenetic aberrations play an important role in acquired cancer drug resistance^[9,10]^. This section briefly describes the common epigenetic mechanisms and their involvement in acquired breast cancer chemoresistance followed by epigenetic regulation of cellular reprogramming events (EMT and CSC phenotype) associated with chemoresistance. Briefly, DNA methylation, post-translational histone modifications and associated chromatin remodeling regulate the epigenetic signaling and the sequential heritable gene expressions.

### DNA methylation, histone modifications and chromatin remodeling

DNA methylation, the most studied epigenetic mechanism in mammals, involves the covalent addition of methyl group to the C5 of cytosine base present exclusively in CpG sites that forms 5 methyl cytosine (5-mC). DNA methyl transferase (DNMTs) enzymes (writers of methylation), using methyl donor S-adenosyl-methionine (SAM), catalyze the methylation of DNA molecules^[44]^. DNA methylation stably silence gene repression through direct inhibition of TFs as well as through recruitment of other group of repressive proteins (methylation readers). These proteins include methyl-binding proteins such as MeCPs, MBDs, zinc-finger domain proteins and UHRF (ubiquitin-like, containing PHD and RING finger domain) proteins, that bind methylated cytosines^[44]^.

Core nucleosome proteins (histones) and their covalent post-translational modifications together dynamically regulate chromatin structure and constitutes the part of the epigenetic regulatory machinery that dictate gene expression^[44,45]^. These epigenetic modifications are combinatorial and occur at histone H3 and H4 moieties through acetylation, methylation, phosphorylation, sumoylation and ubiquitination. Histone acetylation class of enzymes include Histone acetyl transferases (HAT1, Gcn5/PCAF, MYST, p300/CBP, and Rtt109) and Histone deacetylases (classic HDACs and sirtuins HDACs- SIRTs). Histone methylation class of enzymes include Histone methyl transferases (lysine and arginine HMTs- EZHs, MLLs, SETs and PRMTs) and Histone demethylases (HDMTs and KDM1s)^[45]^. Both classes of enzymes dynamically co-ordinate to relax (hyperacetylate) and condense (hypoacetylate) the chromatin and regulate global and/or promoter-specific gene transcription^[45]^ involved in tumorigenesis and chemoresistance^[45,46]^. In addition, these histones modifying enzymes act upon non-histone protein targets to regulate gene expression.

HATs and HMTs catalyze the addition of acetyl and methyl group, respectively, to either lysine or arginine, and HDACs and HDMTs catalyze the removal of acetyl or methyl groups, respectively. For histone methylation, depending upon the amino acid moiety and their site of methylation, gene function can be activated or silenced. Typically, acetylation of H3/H4 in their lysine residue and methylation of H3 at its lysine 4 (H3K4me and H3K4me3) residue mark for transcriptionally active chromatin while methylation of H3 at its Lysine 9 and 27 (H3K9me2 and H3K27me) mark for repressive chromatin. In addition, methylated histones serve as binding site for MBD proteins that co-orchestrate the downstream gene expression.

In concert with DNA methylation and histone modifications, chromatin remodeling complex proteins involve in epigenetic regulation. Among various drivers of chromatin remodeling as reported earlier^[46-48]^, the SWI/SNF complex, nucleosome remodeling factor (NuRF), Mi-2/NuRD (nucleosome remodeling and deacetylase) complex and Polycomb repressor complex (PRCs) are well known to be involved in cellular reprogramming. PRCs directly methylate DNA and methylate histones through HMT named Enhancer of Zeste Homologue 2 (EZH2) to exert their repressive function^[49,50]^. Particularly, BMI-1 containing PRC1 and EZH2/ SUV12 (suppressor of Zeste homolog 12) containing PRC2 complexes are known to involve in chemoresistance^[46-48]^.

### Co-ordination between DNA methylation, histone modifications and chromatin remodeling

With the growing number of studies, the understanding of dynamic epigenetic regulation is also growing, but still it is unclear how the epigenetic events unfold, i.e., whether DNA sequence- associated DNA methylation signals the histone modifications or chromatin-guided initiation serve as signal for DNA methylation process to begin. Nevertheless, it is well established that the epigenetic layers such as DNA methylation and histone modifications cross-talk to exert their effect in a tightly regulated fashion at global and loci-specific levels^[44,45,51]^. DNMTs-induced CpG methylation engage the methyl-CpG-binding machinery proteins (MeCPs, MBDs, UHRFs) bind to the methylated CpG sites. This complex collaborates with catalytically active deacetylases (Sin3 and HDACs) and HMTs which sequentially are recruited to methylated promoter to impose another epigenetic layer that enact histone deacetylation and methylation^[44,45,51]^. HMTs including SUV39H1 and PRMT5 influence the recruitment and stability of DNMTs. It is evident from recent understandings that histones acetylation protects against DNA methylation through insulators (boundary elements). These insulator components in the genes recruit HATs to keep the chromatin relaxed to permit TFs while limiting the DNMTs and sequential binding of MBD protein and Mi-2 deacetylase complex^[45,51]^.

### Aberrant epigenetic changes contribute to acquired chemoresistance

While DNA methylation and histone modifications orchestrate various essential normal cellular physiology, aberration in the epigenetic landscape also contribute to tumorigenesis and resistance development^[8,23,52]^. *In vitro*, *in vivo* and clinical studies, clearly supported that epigenetic aberration mediated through global hypomethylation coupled with localized promoter hyper-methylation and post-translational histone modifications underwrite chemoresistance^[23,53]^.

Aberrant DNA methylation events occur at early stages of tumor development and trigger further genetic/epigenetic changes to contribute to carcinogenesis and resistance induction. Overexpression of DNMTs, elevated percentage of 5-methyl cytosine content and associated hyper-methylation-mediated repressed gene expression, loss of global DNA methylation and associated transcriptional activation have been reported in breast cancer^[23,53,54]^. Likewise, aberrant HDAC1, HAT1, p300 and Suv4-20h2 HMT expression and associated alterations in genome-wide histone modification on H3 and H4 including atypical acetylation (of H3K9, H3K18, H3K56, H4K12 and H4K16), phosphorylation (of H3 serine 10) and methylation (of H3K9 and H4K20) have been reported to occur in naive breast cancer indicating poor prognosis, upon treatment with DNA damaging cytotoxic drugs and chemoresistance^[53,55]^.

Reversible transcriptional changes controlled by the dynamic epigenetic changes causing heterogeneity in a tumor cell population which may serve as a non-genetic source of variation leading to enrichment of chemoresistant cells. Epigenetic deregulation-mediated pathways include several key signaling pathways involving growth/proliferation signaling, microenvironment, EMT-CSC, apoptosis, drug transport, metabolism, DNA damage repair and others^[23]^. With this review focusing on cellular reprogramming, scope of further discussion narrowed down to how the EMT- CSC pathways induce chemoresistance through epigenetic deregulation.

### Aberrant epigenetic changes associated with EMT/CSC-induced chemoresistance

**Figure 2:**
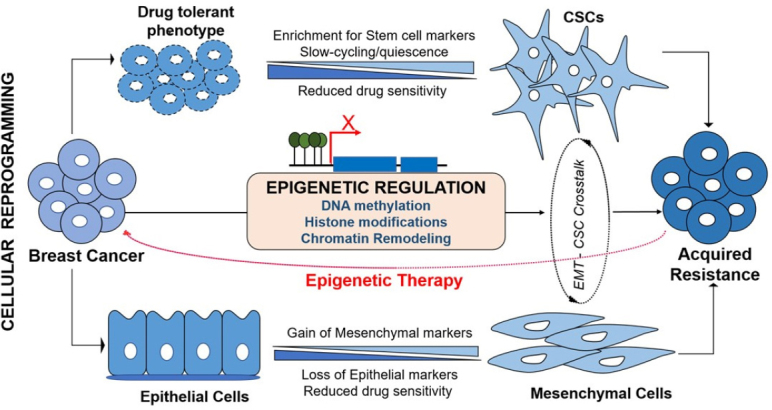
Epigenetic deregulation mediates cellular reprogramming in acquired chemoresistance in breast cancer. Upon exposure to chemotherapy, cancer cells which develop transient (drug tolerant phenotype) may further acquire cancer stem cell (CSC)-like features characterized by enrichment for stem cell markers, slow-cycling and quiescence to facilitate higher levels of resistance development. Similarly, cancer cells preferentially evoke EMT process to evade cytotoxicity. During EMT process, cells with epithelial phenotype progressively loose epithelial markers and gain mesenchymal markers to become aggressive and invasive phenotype. EMT and CSC cross-talk using same signaling pathways to establish acquired resistance. Epigenetic deregulation mediates the acquisition of both EMT and stemness to induce chemoresistance, thus, potential epigenetic therapy targeting the cellular reprogramming could address chemoresistance issues

The reversible nature of EMT and plasticity of CSCs are strategically structured by tight genetic-epigenetic regulation to exert their functional role in chemoresistance^[8]^. These transient changes are regulated through various epigenetic signaling machineries including altered chromatin state and epigenetic modifications. Functional role of DNA methylation and histone modification in establishing the CSC plasticity and differentiation, and epigenetic marks that identifying embryonic as well as cancer stem cells have extensively been reviewed^[6,56,57]^. Deregulation in the DNA methylation and chromatin landscape of both oncogenes as well as tumor suppressors genes initiate the rise of CSCs and EMT. Chemotherapeutic drug-induced DNA-hypermethylation impact the tumor cells response to the cytotoxicity. Concurrently, upon exposure to drug, tumor cells undergo cellular reprogramming to acquire survival-associated phenotypical changes such as EMT and stemness. In addition to key epigenetic events, metabolic components have recently been implicated in altering the plasticity, physiological state and fate of the stem cells. Epigenetic metabolic donors/ factors such as S-adenosyl methionine (SAM), acetyl-CoA, flavin adenine dinucleotide (FAD), nicotinamide adenine nucleotide (NAD+) and α-ketoglutarate influence the DNA methylation and histone modifications to guide the normal and cancer cells towards CSC-like transition^[58]^. These concurrent deregulations that lead to chemoresistance support the likely impact of epigenetic fluctuations in EMT-CSC transduction pathway [Figure 2].

Mounting evidence emphasizes the role of epigenetic deregulation on several genes and pathways that linked with EMT, cancer stemness and acquired resistance. Besides regulation of EMT-CSC genes, aberrant methylation (hypo/hyper) and histone modifications are found to regulate MDR1, MRP2, MRP5, BCRP, Catalase, ALDH1, ESR1, ID4, PITX2, Cyclin D2, P16, P53, Survivin, Bcl2, HIF1α, Leptin, Stromal cell-derived factor receptor 1, MLH1 and MSH2 genes which may provide survival advantage to cytotoxicity^[53,59-61]^. These genes work through distinct mechanisms to provide mechanism-specific drug tolerance, however, may also be activated by concurrent pathways that are associated with EMT-CSCs, hence the co-existence is often observed in resistant population [Figure 2]^[20,53]^.

This section of the review further describes the reported epigenetic alterations on target genes, transcription factors, and signaling pathways that mediate the EMT-CSC pathways and associated acquired resistance in breast cancer. Studies involving genome-wide mapping and global profiling postulate that EMT is characterized by reprogramming of selective chromatin domains across the genome^[53,62,63]^. To begin with, loss of E-Cadherin (CDH1) expression, the initial step in EMT process, shown to occur due to promoter methylation, chromatin modification and associated transcriptional repression^[8]^. For example, loss of E-cadherin and ESR1 due to promoter hypermethylation have been reported in primary breast carcinoma cells^[64]^ and doxorubicin-resistant breast cancer cells^[53]^ and demethylation restored the expression levels of these genes^[64]^. Genome-wide methylation in doxorubicin resistant breast cancer cells found EMT pathway as a critical process for acquisition of resistance^[12]^. Likewise, Chekhun *et al*.^[53]^ revealed that pronounced global epigenetic changes such as loss of global DNA methylation, elevated phosporylation of H3S10, loss of H4K20me3, reduced Suv4-20h2 activity in chemoresistant breast cancer MCF-7^[53]^. In the same study, hypomethylation of Activin A, a negative regulator for E-Cadherin and FOXK1, a positive regulator for Snail-associated EMT were also observed. Correspondingly, the HAT p300 in co-ordination with C-terminal binding protein (CtBP) initiate EMT through the acetylation of SMADs in the TGF-β signaling pathway. Elevated HAT p300 increased vimentin expression and associated with migration, tumor recurrence, and chemoresistance^[65]^. This notion was further supported through depletion p300 that induced epithelial phenotype characterized by decreased vimentin and elevated E-cadherin^[66]^.

Aberrations in EMT-TFs and associated epigenetic changes have been evident in breast cancer acquired resistance. Overexpression of Snail 1, ZEB1, and Twist linked to poor treatment response and acquired resistance in breast cancer through the EMT pathway^[67,68]^. Epigenetic modulations in support of these EMT-TFs indicate that Twist, Snail, Slug, and ZEBs coordinate to recruit chromatin modifying complexes after binding with E-cadherin (CDH1) promoter^[8,68]^. For example, Snail 1 interacts with DNMTs, G9a, Suv39H1, EZH2, and LSD1 to silence the E-cadherin promoter during activation of EMT signaling. Specifically, Snail 1 recruit the repressor complex SIN3/ HDAC1/2 to CDH1 promoter, and coordinates with LSD1 to demethylate the CDH1 followed by interaction with G9a/ Suv39H1 to induce deacetylation of histone H3/H4 and trimethylation of H3K9 to repress E-cadherin expression^[43]^ Similarly, ZEBs interact with CtBP to form ZEB1/p300/PCAF complex that activate ataxia-telangiectasia mutated (ATM) kinase pathway^[68]^. Activated ATM signaling facilitate efficient DNA repair and associated chemoresistance^[68]^. Notably, Twist interact with nucleosome remodeling and deacetylase complex containing Mi2/NuRD-MTA2, RbAp46 and HDAC2 that result in H3K9 deacetylation and H3K9 methylation to mediate promoter-associated silencing of E-cadherin and ER-α^[69]^. These findings further support that epigenetic alterations take part in EMT induction to facilitate resistance development.

Elevated SIRT1 deacetylase modulate the nuclear localization of FOXO1, an apoptosis associated EMT-TF and deacetylate the acetylated DNMT1 thus inhibition of SIRT1 enhance the DNMT1-mediated silencing of ER-α and CDH1 reflecting mesenchymal acquisition in MDA-MB-231 breast cancer cells^[70]^. Overexpression of epigenetic repressor EZH2 involve in EMT, self-renewal potential of CSCs, aggressiveness, poor survival, tumor recurrence and resistance in breast cancer^[71]^. Basal-like breast cancer cells that express mesenchymal characteristics overexpress EZH2, co-ordinates with SUZ12 to form PRC2 complex and recruited to CDH1 promoter to represses E-cadherin^[8]^. ZEB1/2-mediated repression of E-cadherin associated with DNA methylation-regulated silencing of polycomb protein Mel-18^[72]^ to induce EMT in breast cancer cells.

An EMT-associated HMT, G9a, increase *de novo* methylation of CDH1 promoter and loss of its expression^[43]^. The SET8 localized to CDH promoter and methylate H4K20 that causes loss of E-Cadherin and gain of N-Cadherin expression^[73]^. Lysine-specific demethylase-1 (LSD1) caused global reduction in H3K9Me2 and increase in H3K4Me3 and H3K36Me3 during TGF-β-mediated EMT and associated migration and chemoresistance^[62]^. Other mechanisms of LSD1 as to EMT process have been reported while they are inconclusive for breast tumors^[8]^. Moreover, EMT associated miRNAs and their modulation through epigenetic regulation have been reviewed^[8]^. These evidences further underwrite the epigenetic landscape regulating EMT phenotype to cause acquired resistance.

Epigenetic dysregulation triggering formation of CSC phenotype, maintaining their self-renewal and apoptotic resistance to chemotherapy have been accruing. Inactivating mutations of epigenetic regulatory genes including DNMT3A, TET, and PRC2 complex genes aberrantly activate CSC pathways and associated resistance. Similarly, non-mutational epigenetic aberrations of genes KDM5B, G9a/EHMT2, EZH2, PRC1, BMI, BRD4, PRMT5, KDM1A/2A, MLL and SWI/SNF involved in dysregulation of CSC-self-renewal, stemness maintenance and drug sensitivity have been reviewed^[6]^. Gene silencing mediated through DNMTs, EZH2/BMI1 and HDAC1, and activation through MLL and CBP potentially induce aberrations associated with BCRP, E-cadherin, p16 and Wnt signaling in CSCs^[52]^. Epigenetic alterations evident to induce intratumoral heterogeneity through modulating microenvironment and associated signaling which supports CSCs including Wnt, TGF-β, and Notch signaling^[6,23]^. Transient drug-tolerant states generated by reversible poised chromatin state are regulated through histone demethylase RBP2/KDM5A/Jarid1A and Notch signaling, thus, help CSCs to evade cytotoxicity mainly by equipping them with complex resistance mechanisms^[22]^.

Similar to epigenetic regulating proteins, the TFs associated with EMT and CSC work coordinately to activate the downstream effects related to drug resistance. Cancer cells with stemness known to express high levels of metastatic markers such as Snail1, Twist 1 and FOXC2. Elevated FOXC2 in basal-like breast cancer cells activate EMT-CSC signaling and reduce drug sensitivity^[74]^. Similarly, overexpression of Twist selects cells with CD44-high/ CD24-low subpopulation that have increased MRP1 expression and activate β-catenin/Akt pathways to maintain stemness^[75]^. Similarly, Stem cell factor BMI-1, which is also part of PRC1 complex reportedly involve in chemoresistance^[76]^. Twist 1 directly activates the BMI1 expression and they together orchestrate the EMT and stemness induction and maintenance. BMI-1 also shown to regulate stem cell renewal and induce EMT through its co-ordination with Nanog and NF-κB pathway^[77]^. Meanwhile loss of BMI-1 increased sensitivity of breast cancer cells to doxorubicin^[77]^.

Epigenetic deregulation associated with signaling pathways involved in EMT-CSC co-ordination further implicate their roles in chemoresistance. For example, Wnt ligands and sequester proteins including Wnt5A, WIF-1, FZD and SFRP known to be modulated through promoter methylation and histone modifications in breast cancer^[39]^. Aberrations in epigenetic regulations evaluating TGF-β, Hh and Notch signaling in CSCs are scarce. Hh signaling ligands SHH, PTCH1 and SOX17 frequently methylated in breast cancer initiating cells and Hh effector proteins KIF7 and SUF7 deregulated through histone modifications and miRNA while GLI1 expression is elevated due to loss of KMT-SETD7 methylase in breast cancer cells thus drive the aberrant Hh as well as associated NF-κB signaling^[39]^. Epigenetic mechanisms regulating Notch signaling occur through HES, a Notch effector and transcriptional repressor, which recruit histone acetylases/deacetylases and through hypermethylation mediated silencing of DLL1 (Notch ligand). Taken together, all these evidences implicate the epigenetic aberrations dictating the activation of EMT-CSC pathways in acquired chemoresistance in breast cancer.

### Impact of treatment schedule on resistant development through acquisition of EMT and CSC-like phenotype and associated temporal epigenetic changes

In support of the reports discussed in previous section, we are highlighting, in this section, the recent findings from our laboratory demonstrating the influence of treatment schedules on acquired chemoresistance in breast cancer cells through epigenetic aberrations. During acquired resistance development to doxorubicin, significant morphological changes with acquisition of EMT phenotype and/or CSC-like growth properties are reported only in intermittently treated breast cancer cells (MCF-7 and MDA-MB-231 cells)^[37]^. In this study, MCF-7 cells that are typically polarized epithelial cells with cuboidal to columnar shape acquired elongated spindle shaped mesenchymal morphology upon intermittent exposure to doxorubicin. More interestingly, the degree of acquired EMT phenotype was increased with treatment and correlated with level of acquired resistance to doxorubicin. With similar treatment, MDA-MB-231 cells which are originally mesenchymal in origin, acquired further enhanced mesenchymal phenotype. These changes accompanied with down-regulation of epithelial markers, elevated mesenchymal promoting transcription factor FoxC2, repressor transcription factor of E-cadherin Snail 1, and mesenchymal markers (N-cadherin, vimentin and fibronectin).

Besides, accompanied the EMT phenotype were cells with CSC-like characteristics with formation of free-floating colonies (tumorospheres) upon intermittent doxorubicin treatment. These features uniquely noted in MCF-7 cells and corresponded with relatively early and higher level of resistance developed. Interestingly, in this published study, acquisition of EMT and CSC phenotypes were observed only in intermittently exposed cells and not in continuously exposed cells. In a continuation study^[78]^, when the treatment continued for 18 months, the acquired resistant cells were enriched for CSC and metastasis markers (ALDH1A1, CD44, Nanog, OCT4, MMP2 and VEGFA) and acquired increased tumorigenicity and resistance to therapy^[78]^. This study further tracked the transcriptional level epigenetic changes in these cells after 3 months (when cells acquired pronounced EMT and CSC-like phenotype) and after 18 months (when 30-fold increase in resistance observed with enrichment for CSC-markers) and found temporal increase in epigenetic markers expression including DNMT1, DNMT3a, DNMT3b, and HMT1^[78]^. DNA demethylating agent 5-Aza-2’-deoxycytidine significantly inhibited the tumorigenicity, tumorospheres formation and sensitized resistant cells to doxorubicin. These recent reports along with other evidences further support the role of cellular reprogramming through epigenetic changes involves in acquired chemoresistance.

### Epigenetic therapy focused on cellular reprogramming associated with acquired chemoresistance

Reversible and dynamic nature of the epigenetic aberrations create potential opportunities in terms of therapy. Epigenetic modifying drugs are potential therapy approach mainly because they target epigenetic enzymes that regulate cell’s genetic programming rather than targeting cancer cells as such. Given the fact that cellular programming initiates the transient drug tolerance that further lead to resistance development, addressing cellular programming is crucial. Targeting epigenetic landscape of cancer stem cells as potential therapeutic intervention have been explored for different cancer subtypes^[79]^. Since CSC cells enter quiescent state, inducing cell differentiation from their quiescent states seem promising approach to resensitize the resistant cells to chemotherapy. This can be achieved using DNA demethylating agents, histone deacetylase (HDAC) inhibitors, histone methyltransferase (HMT) inhibitors and histone demethylase (HDM) inhibitors.

Currently, the epigenetic treatment approaches addressing the EMT-CSC pathway are experimental, and clinical investigations thus far are scarce. Potential epigenetic reprogramming target to address EMT-CSC include the Twist-Snail-E-Cadherin-ZEB axis, Wnt-TGF-β-BMI axis, and Wnt-TGF-β-SOX axis. Accumulating evidence suggests that the effect of DNA demethylating agents and HDAC inhibitors either alone or in combination with chemotherapeutic drugs to resensitize chemoresistance works through cellular reprogramming^[6,78,80]^. Besides reactivating silenced tumor suppressor genes and inducing differentiation of CSCs, the low dose DNA methylating agents and HDAC/ HMT/ HDM inhibitors reduce tumorigenicity and prevent CSC invasiveness and tumor metastasis^[79]^.

Several epigenetic modifying agents are under clinical trial investigations [Tables 1 and 2] while few of the them have been approved for treatment^[52,80,81]^. Additionally, there are current clinical trials undergoing specific to breast cancer including epigenetic and non-epigenetic therapies that work through pathways potentially associated with cellular reprogramming-EMT/CSC [Table 1]. Evidence from preclinical studies of these drugs on EMT/CSC inhibition have been reported in other cancers [Table 1]. With respect to breast cancer resistance, addressing the heterogeneity is essential^[3,23]^ which warrants epigenome profiling of resistant tumors in order to identify the potential targets or silenced marker genes before employing epigenetic drugs. Documenting the epigenetic landscape and exploring it further to understand the changing epigenome are inevitable to assess the clinical efficacy of epigenetic modifiers either solely or priming with existing chemotherapeutic drugs. Moreover, combination of drugs that address DNA methylation and histone modifications may act synergistically to regulate the cross-talk between DNA methylation and histone modification machineries.

**Table 1:** Epigenetic and non-epigenetic agents under clinical trial for breast cancer and their mechanism associated with cellular reprogramming

Drugs	Type of breast cancer	Clinical trial ID	Cellular reprogramming pathways targeted by these drugs	Ref.
Non-epigenetic modifying agents				
GDC - 0084 + Trastuzumab	Her2 +ve BC	NCT03765983	PI3 - Akt pathway	[30]
Ganetespib + Paclitaxel	TNBC	NCT02637375	HSP90 inhibition; PI3-Akt pathway	[82]
BIIB021	MBC	NCT01004081	HSP90 inhibition	[82]
Gedatolisib	TNBC	NCT03243331	PI3K/mTOR pathway	[27]
BYL719 + Nab-Paclitaxel	Her2 -ve BC	NCT02379247	PI3K pathway	[30]
BMS - 754807	Her2 +ve BC	NCT00788333	IGF-1 inhibition	[83]
Epigenetic modifying agents				
Entinostat + Capecitabine	Metastatic BC	NCT03473639	HDAC inhibition	[80,81]
Entinostat + Exemestane	Metastatic BC	NCT02833155		
Hydralazine-Mg Valproate	Chemotherapy Resistance Solid Tumors	NCT00404508		
Panobinostat (LBH589)	Tamoxifen refractory BC	NCT00993642		
Vorinostat + Olaparib	MBC	NCT03742245		

BC: breast cancer; TNBC: triple negative breast cancer; MBC: metastatic breast cancer; mTOR: mammalian target of rapamycin; HDAC: istone deacetylases; HSP: heat shock protein; IGF-1: insulin-like growth factor 1

**Table 2:** Epigenetic modifying agents either approved or under clinical trial for different cancer types and their mechanism associated with cellular reprogramming

Drugs	Type of cancer	Cellular reprogramming pathways targeted by these drugs	Ref.
Azacytidine, 5-Aza-2’-deoxycytidine	Refractory solid tumors; Head and neck squamous carcinoma; Hematological; Ovarian, Prostate, Lung and Colorectal cancers	DNMT inhibition	[80,81]
FK228, MGCD0103 and Entinostat		Class I HDAC inhibition	
Vorinostat, Pracinostat, Panobinostat, Belinostat, ITF2357, PCI-24781, LAQ824, Phenyl butyrate, Valproic acid and Trichostatin A		Pan-HDAC inhibition	
Nicotinamide, Cambinol, Tenovin 1, Tenovin 6, Sirtinol and EX-527		Class III SIRT1 inhibition	
Mocetinostat		Class I/IV HDAC inhibition	
Tozasertib, Danusertib, AZD1152, AS703569, AT9283 and SNS-314		Aurora-B kinase inhibition	

DNMT: DNA methyl transferases; HDAC: histone deacetylases

## Future direction and conclusion

Acquired chemoresistance in general involves a broad range of poorly-comprehended domains (genetic-epigenetic) and different partners that cross-talk within and between domains that underwrite the need for an integrated approach. Strong body of evidence exists to support the role of epigenetic changes that contribute to cellular reprogramming (leading to EMT and CSC phenotype) during acquired resistance development. Though the existing reports support the functional role of EMT and associated signaling in cancer stemness, the question of whether EMT solely drive stemness to induce chemoresistance in the face of co-existing genetic-epigenetic landscape remain to be explored. Nevertheless, addressing epigenetic regulation associated with cellular reprogramming is important in the context of chemo-resistance and breast cancer treatment. A better understanding of the dynamic epigenetic regulatory network in initiating and stabilizing the EMT-CSC signaling in breast cancer is needed. This will help designing better therapies that address not only the genetic changes but also the reversible epigenetic changes during the transient process of EMT and stemness.

## Declarations

### Copyright

© The Author(s) 2019.
